# Promising System for Selecting Healthy *In Vitro*–Fertilized Embryos in Cattle

**DOI:** 10.1371/journal.pone.0036627

**Published:** 2012-05-09

**Authors:** Satoshi Sugimura, Tomonori Akai, Yutaka Hashiyada, Tamás Somfai, Yasushi Inaba, Muneyuki Hirayama, Tadayuki Yamanouchi, Hideo Matsuda, Shuji Kobayashi, Yoshio Aikawa, Masaki Ohtake, Eiji Kobayashi, Kazuyuki Konishi, Kei Imai

**Affiliations:** 1 National Livestock Breeding Center, Nishigo, Fukushima, Japan; 2 Dai Nippon Printing Co., Ltd., Kashiwa, Chiba, Japan; 3 National Institute of Livestock and Grassland Science, Tsukuba, Ibaraki, Japan; University of Connecticut, United States of America

## Abstract

Conventionally, *in vitro*–fertilized (IVF) bovine embryos are morphologically evaluated at the time of embryo transfer to select those that are likely to establish a pregnancy. This method is, however, subjective and results in unreliable selection. Here we describe a novel selection system for IVF bovine blastocysts for transfer that traces the development of individual embryos with time-lapse cinematography in our developed microwell culture dish and analyzes embryonic metabolism. The system can noninvasively identify prognostic factors that reflect not only blastocyst qualities detected with histological, cytogenetic, and molecular analysis but also viability after transfer. By assessing a combination of identified prognostic factors—(i) timing of the first cleavage; (ii) number of blastomeres at the end of the first cleavage; (iii) presence or absence of multiple fragments at the end of the first cleavage; (iv) number of blastomeres at the onset of lag-phase, which results in temporary developmental arrest during the fourth or fifth cell cycle; and (v) oxygen consumption at the blastocyst stage—pregnancy success could be accurately predicted (78.9%). The conventional method or individual prognostic factors could not accurately predict pregnancy. No newborn calves showed neonatal overgrowth or death. Our results demonstrate that these five predictors and our system could provide objective and reliable selection of healthy IVF bovine embryos.

## Introduction

Conventionally, the criteria for selecting bovine *in vitro fertilized* (IVF) embryos for transfer are based on morphological quality at the time of transfer [1]. However, this approach is widely considered extremely subjective and inadequate [2]. Furthermore, the pregnancy outcomes of blastocysts estimated by evaluators to be morphologically good to excellent remain low (37–52%) [3]. Therefore, novel criteria allowing objective and reliable selection of embryos for transfer are required to advance bovine IVF technology.

Aside from morphological quality, embryonic cell number of the inner cell mass (ICM) and trophectoderm (TE) [4], apoptosis incidence [5], hatching competence [6], chromosomal abnormalities [7], and expression of specific genes [8] have been widely accepted for determining embryo quality. Histological, cytogenetic, and epigenetic analyses do, however, make transferring the embryo to a recipient more difficult after these analyses and may also make practical embryo selection more cumbersome and complicated. Noninvasive criteria that could predict not only blastocyst qualities but also viability may therefore lead to novel methods for selecting bovine embryos for transfer.

Following ovum pickup (OPU), which collects a limited number of oocytes, culture system for undergoing *in vitro* oocyte maturation (IVM), IVF and embryo culture (IVC) individually appear to be practical [9]. Recently, we developed a microwell culture dish based on the well-of-the-well system [10], which allows tracking of individual embryo with time-lapse cinematography (TLC) observation [11]. TLC is an effective method for continuous imaging of embryo developmet *in vitro*, allowing analysis of the developmental kinetics, blastomere number, symmetry of cell division, and the extent of cytoplasmic fragmentation, which have been used to select the best embryos for transfer in human assisted reproductive technologies [12]–[14]. In cattle, these criteria are possible predictors for developmental competence to blastocyst stage [15], [16], but rarely used to selecting embryos for transfer.

In addition to the studies using TLC, measurement of embryo metabolism parameters such as oxygen consumption has attracted attention in predicting embryonic development. Using modified scanning electrochemical microscopy (SECM), which appears to be a reliable, noninvasive, and highly sensitive method for measuring oxygen consumption in individual embryos [17]–[19]. However, directly assess embryo viability, as embryo transfer to recipients following these measurements was limited performed [20].

Here we sought to identify various prognostic factors that allow prediction of bovine blastocyst qualities such as embryonic cell number, apoptosis incidence, hatchability, chromosome abnormalities, and gene expression using a combination of TLC imaging with microwell culture dish and oxygen consumption measurement. Subsequently, to examine the practical advantage of the identified prognostic factors for predicting post-transfer viability, OPU-IVF–derived blastocysts that were assessed by individual or multiple factors were transferred to recipient cows.

## Materials and Methods

### Ethics statement

This study was approved by the Ethics Committee for the Care and Use of Experimental Animals at the National Livestock Breeding Center located in Nishigo, Fukushima, Japan. All animals received humane care according to law no. 105 and notification no. 6 of the Japanese Guidelines for Animal Care and Use. Bovine ovaries were collected from Omiya abattoir, a local slaughterhouse Omiya, Saitama, Japan. Frozen sperm were obtained from National Livestock Breeding Center Ohu Station located in Shichinohe, Aomori, Japan.

### Oocyte collection and IVM

Collection of bovine cumulus-oocyte complexes (COCs) was performed as described [21]. Ovaries from Japanese Black or Holstein breeds were collected at a local slaughterhouse, transported to the laboratory, washed, and stored in physiological saline supplemented with 50 µg/ml gentamycin (Sigma Chemical, St. Louis, MO, USA) at 20°C for ∼20 h. COCs were aspirated from small follicles (2–6 mm in diameter) using a 5-ml syringe equipped with a 19-gauge needle. The IVM medium was 25 mM HEPES-buffered TCM199 (M199; Gibco, Paisley, Scotland, UK) supplemented with 5% calf serum (CS; Gibco). COCs were washed twice with IVM medium and incubated in 600 µl IVM medium covered with paraffin oil (Paraffin Liquid; Nacalai Tesque, Inc., Kyoto, Japan) in 35-mm Petri dishes (Nunclon Multidishes; Nalge Nunc International, Roskilde, Denmark) at 38.5°C in a humidified atmosphere of 5% CO_2_ in air for 22 h (60–80/droplet).

### IVF

IVF was carried out as reported by Imai et al. [21]. Briefly, at the end of IVM, ejaculated sperm samples from a Japanese Black bull that were frozen in 0.5-ml straws were thawed in a 37°C water bath for 30 s and then centrifuged in 3 ml 90% Percoll solution (GE Healthcare, Uppsala, Sweden) at 750× *g* for 10 min. The pellet was then re-suspended and centrifuged in 6 ml sperm-washing medium (Brackett and Oliphant solution, BO) [22], supplemented with 10 mM hypotaurine (Sigma) and 4 U/mL heparin (Novo-Heparin Injection 1000; Aventis Pharma Ltd., Tokyo, Japan), at 550× *g*. Then the pellet was re-suspended in sperm-washing medium and BO solution supplemented with 20 mg/ml BSA (crystallized and lyophilized; Sigma) to achieve a final concentration of 3×10^6^ spermatozoa/ml. Droplets of this suspension (100 µl each) placed in 35-mm dishes and covered with paraffin oil served as fertilization droplets. COCs were removed from the maturation medium, washed twice in BO supplemented with 10 mg/ml BSA, placed in fertilization droplets (20 COCs/droplet), and cultured for 6 h at 38.5°C in 5% CO_2_ in air with saturated humidity.

**Figure 1:**
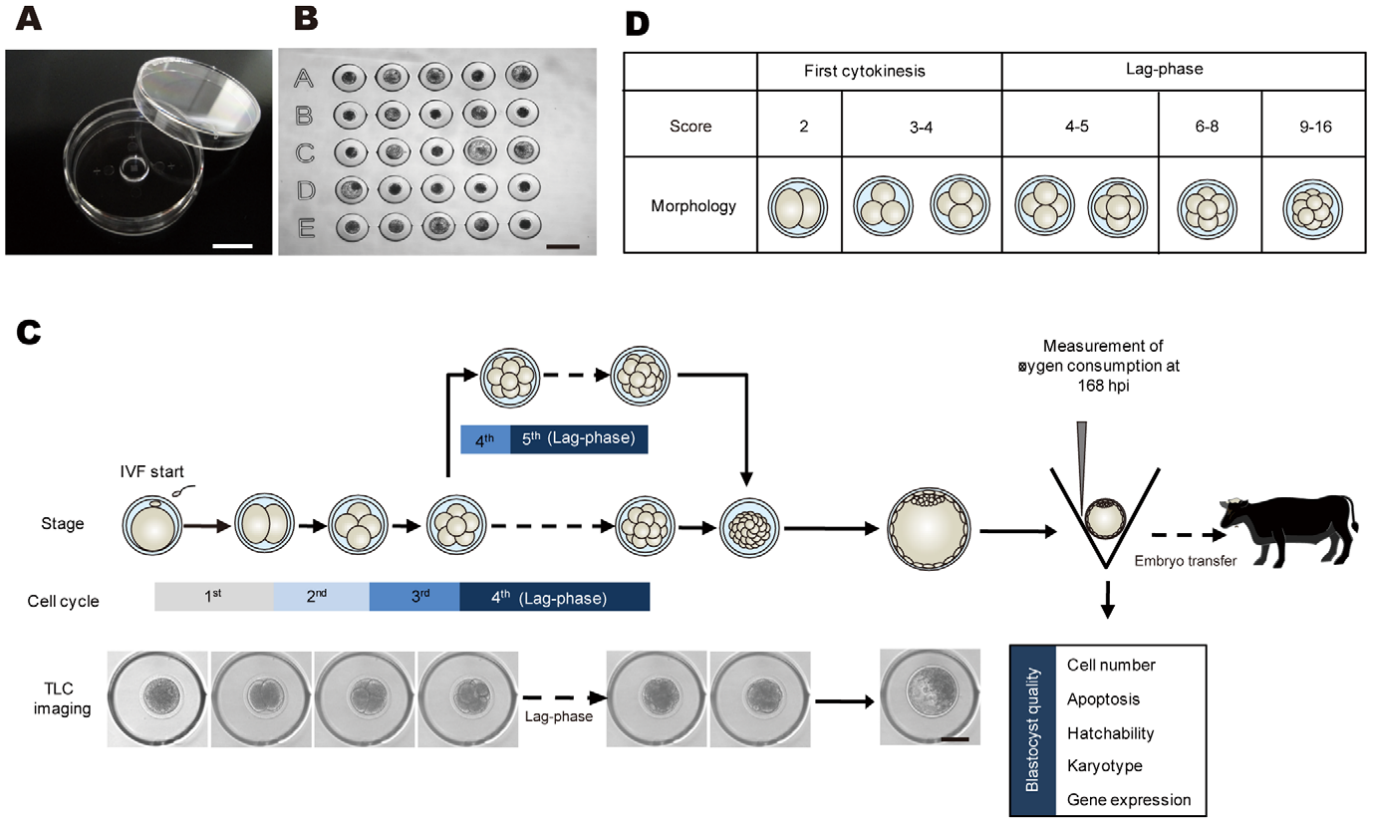
Overview of the experimental scheme. *In vitro* development of bovine IVF embryos in the developed microwell culture dish with identification code (**A** and **B**) was tracked with time-lapse cinematography (TLC) for 168 h post-insemination (hpi). SECM was used to measure oxygen consumption in embryos that developed to the blastocyst stage at 168 hpi (**C**). Timing of the of the first cleavage; duration of the second, third, and lag-phases resulting in developmental arrest at the fourth or fifth cell cycle; number of blastomeres; presence or absence of multiple fragments; unevenness or evenness of division at the end of the first cleavage and at the onset of the lag-phase; cell cycle observed lag-phase (fourth or fifth cell cycle); and oxygen consumption at 168 hpi were examined in relation to blastocyst qualities such as cell number (n = 173), apoptosis incidence (n = 74), hatchability (n = 195), karyotype (n = 111), and gene expression (n = 75) with multivariate analysis. Based on the identified prognostic factors that reflected the various blastocyst qualities, OPU-IVF blastocysts (n = 52) were then assessed and transferred to recipient cows (n = 52). (**D**) The number of blastomeres at the end of the first cleavage was categorized as 2 and 3/4 blastomeres. The numbers of blastomeres at the onset of the lag-phase were categorized as 4/5, 6–8, and 9–16 based on the result from Fig. S1. Bar = 10 mm (**A**), 300 µm (**B**) and 100 µm (**C**).

### Design and fabrication of the microwell culture dish

The microwell culture dish (Dai Nippon Printing Co., Ltd., Kashiwa, Japan) was designed and fabricated as described [11]. The dish has 25 microwells with identification (ID) codes and a circular wall in the center of a 35-mm culture dish. Each well is 287 µm in diameter and 168 µm deep, and the 25 wells are arranged in five columns and five rows. The wells are 150 µm apart. The bottom of each well slopes down toward the center of the well (slope angle is 7.1°). The circular wall is 7 mm in diameter and 1.5 mm in height and is used to form a microdroplet of culture medium. ID codes A to E and 1 to 5 are tagged on the left and top of the microwells, respectively. The microwell culture dish was fabricated by the conventional injection molding method. First, a metal mold was fabricated by machining, and then the microwell culture dish was replicated by an injection molding machine with the fabricated mold. Polystyrene was chosen as the material for the microwell culture dish because of its non-toxicity in cell culture.

### Preparation of the microwell culture dish

Preparation of the microwell culture dish was performed as described [11]. Ethanol (2 ml) was added to each microwell culture dish for washing and sterilization. After 30 min, the ethanol was removed, and the microwell culture dish was air dried on a warm plate and ultraviolet-sterilized for 10 min. After sterilization, 125 µl Charles Rosenkrans 1 with amino acid (CR1aa) medium [23] supplemented with 5% CS was placed within the circular wall and covered with paraffin oil. Air bubbles inside the microwells were flushed out by tapping the dish wall from outside with an awl. The microwell culture dish was pre-incubated for at least 3 h before use.

### IVC

IVC was performed at 38.5°C in 5% O_2_, 5% CO_2_, and 90% N_2_ with saturated humidity for 168 h in the prepared microwell culture dish. After insemination, putative zygotes were completely denuded from cumulus cells and spermatozoa by gentle pipetting with a fine glass pipette in pre-incubated IVC medium. Twenty-five zygotes were placed in microwells of the microwell culture dish (one zygote per microwell).

### TLC Imaging

TLC imaging was evaluated as reported [24]. *In vitro* development was monitored with a Real-Time Cultured Cell Monitoring System with Multiple-Point Imaging Capture (CCM-MULTI; Astec, Fukuoka, Japan). During the 168-h culture period, 673 photographs of the embryos were taken at 15-min intervals using a plan objective with 4× magnification. Image stacks were analyzed using CCM ver. 2.1.0.4 software (Astec). The time of the first appearance of the following cleavage or embryo stages was recorded for zygotes in focus with identifiable blastomeres: 2-, 3- or 4-, 5- to 8-, and 9- to 16-cell stages and for morulae and blastocysts. If proper evaluation of specific cleavage events was not possible, the particular data points were treated as missing data. The cleavage stages of embryos in which one or more blastomeres stopped further cleavage were categorized according to the number of cell cycles observed in the healthy blastomeres. From the fourth cell cycle onwards, individual blastomeres could not be observed directly. However, passing from the fourth and fifth cell cycles to the following cycle was defined by combining direct chronological observation of cleaving blastomeres or the initiation of intensive movements within the cell mass created by blastomere cleavage, the cessation of all cleavages and movements followed by a resting period, and the start of a new cleavage round creating new movements. The presence of a morula was defined as when the first signs of compaction could be observed, but with blastomeres still clearly distinguishable on the surface. The appearance of the blastocyst stage was characterized by the first appearance of a stable, confluent blastocoel. Finally, blastocysts were characterized as expanded when the thickness of the zona pellucida was decreased as a result of expansion of the blastocoel.

### Measurement of oxygen consumption

Oxygen consumption by individual bovine embryos was measured non-invasively with a SECM system (HV-405SP; Hokuto Denko Co., Tokyo, Japan). Embryos were individually transferred to a plate filled with 5 ml embryo respiration assay medium-2 (ERAM-2; Research Institute for the Functional Peptides, Yamagata, Japan), and the embryos were dropped individually to the bottom of the microwell. The temperature of the medium was maintained at 38.5°C by placing the plate on a warming plate on the microscope stage. Oxygen consumption was measured as described by Shiku et al. [17]. The XYZ-stage and the potentiostat were controlled by HV-405 ver. 2.04 (Hokuto Denko). Voltammetry of the Pt-microdisc electrode (Hokuto Denko) in ERAM-2 solution showed a steady-state oxygen reduction wave. No response from other electrochemically active species was observed near the embryo surface. The oxygen consumption rate of embryos was calculated using HV-405 ver. 2.04. The oxygen concentration difference between the bulk solution and the sample surface (Δ*C*) and the oxygen consumption rate (*F*) of a single sample were estimated according to spherical diffusion theories [17]. We repeatedly scanned the electrode back and forth to estimate the mean Δ*C* for each sample two times.

**Figure 2:**
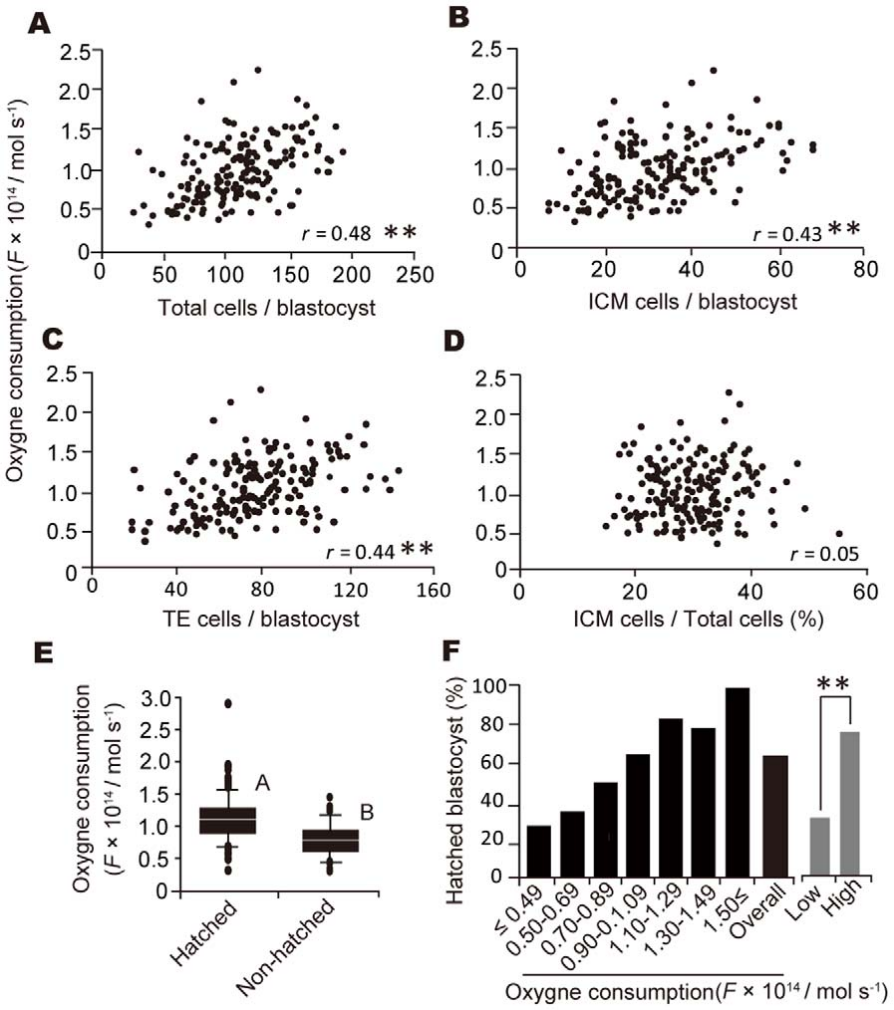
Correlation between oxygen consumption at the blastocyst stage and number of total (A), ICM (B), and TE (C) cells and the percentage of ICM cells relative to total cell number (A4). **Coefficient of determination, *r*, was statistically significant based on simple regression analysis (*P*<0.01). Relationship between oxygen consumption at the blastocyst stage and hatching after 48 additional hours of culture (**D** and **E**). The level of oxygen consumption was considered high or low with a cut-off of 0.84×10^−14^ mol s^−1^ (**E**). Double asterisks indicate significant differences between groups based on the χ^2^ test (*P*<0.01).

### Differential staining of ICM and TE cells

The cellular composition of blastocysts was assessed by differential staining of ICM and TE cells as described by Thouas et al. [25]. Briefly, TE cells of blastocysts were stained with 100 µg/ml propidium iodide (PI; Sigma) fluorochrome containing a permeabilizing solution of 0.2% (v/v) Triton X-100 ionic detergent (Sigma) for 40 s. Blastocysts were then incubated for 5 min in a second solution of 25 µg/ml Hoechst 33342 (Calbiochem, La Jolla, CA, USA) in 99.5% ethanol for fixation. Fixed and stained whole blastocysts were mounted and assessed for cell number using an epifluorescence microscope (IX-71; Olympus, Tokyo, Japan). ICM and TE nuclei were blue and pink-red, respectively.

### Detection of apoptosis-positive cells with the TUNEL assay

Detection of apoptosis-positive cells with the TUNEL assay was performed as described by Yamanaka et al. [26]. Blastocysts were washed three times in PBS supplemented with 0.1% polyvinylalcohol (PVA; Sigma) and fixed in 2% (w/v) paraformaldehyde (Sigma) and 0.2% (v/v) Triton X-100 in PBS for 40 h at room temperature. A commercially available kit (ApopTag; Chemicon, Temecula, CA, USA) was used to detect apoptosis-positive cells. For the positive control, blastocysts were treated with DNase I (10 IU/mL, Sigma). After being washed three times with 0.1% PVA in PBS for 10 min each, blastocysts were incubated in the equilibration buffer from the kit for 20 s at room temperature. They were then incubated at 37°C for 2 h in a moist chamber in 30% (v/v) terminal deoxynucleotidyl transferase in reaction buffer. The reaction was stopped by adding 3% (v/v) stop/wash buffer (Chemicon) at 37°C for 10 min. After washing four times with PBS containing 0.2% (v/v) Triton X-100 for 2 min each, they were incubated with anti-digoxigenin antibody conjugated to horseradish peroxidase at room temperature for 1 h. After washing four times with 0.2% Triton X-100 and 0.1% PVA in PBS, blastocysts were stained with 10 µg/mL PI in PBS for 1 h at room temperature. All samples were examined under an epifluorescence microscope. TUNEL-positive nuclei were yellow-green. The apoptosis incidence of the embryos was calculated as the percentage of TUNEL-positive nuclei relative to the total number of nuclei.

### Evaluation of hatchability from the zona pellucida

To assess subsequent development *in vitro* after the initial 168-h incubation, individual embryos were transferred to 20-µl droplets of CR1aa supplemented with 5% CS and covered with paraffin oil in 35-mm dishes (one blastocyst per drop). Incubation was performed at 38.5°C in 5% O_2_, 5% CO_2_, and 90% N_2_. Hatching (i.e., complete emergence from the zona pellucida) was assessed after 48 additional hours of culture.

### Chromosomal preparation for karyotyping

Chromosome samples of embryos were prepared as described by Somfai et al. [24]. Blastocysts were cultured for 14–17 h in CR1aa supplemented with 5% CS and 60 ng/ml vinblastine sulfate (Wako Pure Chemical Industries, Osaka, Japan). The blastocysts were then washed and incubated in 1% (w/v) sodium citrate solution for 15 min and fixed by pouring 0.02 ml acetic methanol (1∶1 (v/v) acetic acid/methanol) into 0.4 ml of a hypotonic solution of sodium citrate. A blastocyst was placed on a glass slide in air with saturated humidity, immediately covered with a very small droplet of acetic acid to separate each cell, and then re-fixed with acetic alcohol (1∶3 (v/v) acetic acid/methanol). After complete drying, chromosome samples were stained with 2% (w/w) Giemsa in distilled water (Merck KGaA, Darmstadt, Germany) for 10 min.

### Cytogenetic criteria

Only cell nuclei that were intact and non-overlapping were analyzed. For each embryo, the total number of cells (interphase+mitosis) was counted, and all analyzable metaphases were examined under oil-immersion bright-field microscopy at a magnification of 1000×(100×10) to determine the chromosome number. Embryos in which all nuclei analyzed contained two sets of chromosomes (2n = 60) or one set of chromosomes (n = 30) were scored as diploid or haploid, respectively, and those in which all nuclei contained 3n, 4n, or ≥5n were considered polyploid. Embryos containing a mixture of diploid cells and cells with more or fewer than two sets of chromosomes were considered mixoploid. The mitotic index was calculated as the ratio between the number of mitotic cells and the total number of cells (interphase+mitotic). Digital images of each chromosome were taken, and numbers of chromosomes were analyzed using NIH Image J (v. 1.40) software.

### Quantitative real-time RT-PCR analysis

Six genes were analyzed with real-time PCR as described [18]: histone H2Az (*H2AFZ*), caudal type homeobox 2 (*CDX2*), interferon tau (*IFNΤ*), placenta-specific 8 (*PLAC8*), aldo-keto reductase family 1 member B1 (*AKR1B1*), and insulin-like growth factor 2 receptor (*IGF2R*). Individual blastocysts were lysed in 50 µl extraction buffer (Arcturus, Carlsbad, CA, USA), incubated at 42°C for 30 min, and stored at −80°C. Total RNA was extracted from each sample using a PicoPure RNA Isolation Kit (Arcturus). Residual genomic DNA was removed with recombinant RNase-free DNase I (Roche, Mannheim, Germany). RNA was eluted from the purification column using 11 µl elution buffer (Arcturus). RNA was reverse transcribed into cDNA using a ReverTra Ace qPCR RT Kit (Toyobo Bio, Osaka, Japan). Each 20-µl reaction product was diluted with nuclease-free water to a final volume of 40 µl. Quantitative real-time RT-PCR was performed using StepOnePlus™ Systems (Applied Biosystems, Foster City, CA, USA) in a 20-µl reaction volume containing 2 µl cDNA, 0.5 µl each of the forward and reverse primers (Table S1), 7 µl nuclease-free water, and 10 µl Fast SYBR Green PCR Master Mix (Applied Biosystems). In each set of PCR reactions, duplicate cDNA samples were run to control for the reproducibility of the real-time RT-PCR results. Universal thermal cycling parameters (initial step of 20 s at 95°C, followed by 45 cycles of 3 s at 95°C, 10 s at 60°C, and 20 s at 72°C) were used to quantify the expression of each gene. After the end of the last cycle, a melting curve program was run. A standard curve was generated for both the target and the endogenous control gene in every PCR run using serial 10-fold dilutions of amplified cDNA derived from blastocysts. Final quantitative analysis was performed with the relative standard method, and results were reported as the relative expression after normalization of the transcript amounts to *H2AFZ* (the endogenous control gene). The quality of the PCR products was confirmed with melting curve analysis.

### Collection of *in vivo*–derived blastocysts

*In vivo*–derived embryos were collected as described by Hashiyada et al. [27]. *In vivo* embryos were collected from superovulated Japanese Black cattle. Superovulation was performed by injecting 20 A.U. follicle-stimulating hormone (ANTORIN®-R•10; Kawasaki Pharmaceutical, Kawasaki, Japan) and 2 ml prostaglandin F_2α_ (RESIPRON®-C; ASUKA Pharmaceutical, Tokyo, Japan) followed by artificial insemination (AI). Semen was the same as that used for production of *in vitro* embryo. On day 8 after AI, embryos were recovered by uterine flushing. Blastocysts and expanded blastocyst-stage embryos were selected and stored in Dulbecco's PBS (D-PBS; Gibco, Grand Island, NY, USA) containing 20% CS.

### Production of embryos derived from oocytes collected by OPU

As described by Imai et al. [21], COCs were collected from Japanese Black cows by OPU, using an ultrasound scanner (SSD-900; ALOKA, Tokyo, Japan) and a 7.5-MHz convex-array transducer (UST-9109P-7.5; ALOKA) with a 17-gauge stainless steel needle guide. Follicles >2 mm in diameter were aspirated with a vacuum (120 mmHg and 22 mL/min aspiration rate) through a disposable aspiration needle (COVA Needle; Misawa Medical, Tokyo, Japan). Oocytes were matured in 50-µl droplets of TCM199 supplemented with 5% CS and fertilized with semen from a proven bull as described above. Following IVF, 10 to 25 embryos were cultured in a microwell culture dish contained 125 µl CR1aa supplemented with 5% CS.

### Synchronization, embryo transfer, and pregnancy diagnosis

Japanese Black/Holstein cross-bred recipients were synchronized with 3 ml prostaglandin F_2α_. A single blastocyst was selected 7 days after insemination and transferred into the ipsilateral uterine horn of each synchronized recipient on day 7 to 8 after estrus. The recipients were fed and managed in the same manner, and estrous behavior was observed at least twice daily, in the morning and evening. Pregnancy diagnosis was performed on day 30 after embryo transfer using ultrasonography (HS101V; Honda Electronics, Toyohashi, Japan). Pregnancy was confirmed by observation of a fetus with a detectable heartbeat in the intraluminal uterine fluid and of an embryonic membrane.

### Identification of variables reflecting blastocyst quality

From data of TLC imaging and oxygen consumption (Fig. 1), variables reflecting several blastocyst qualities were identified with multiple or logistic regression analysis [28]. Potential variables reflecting the embryonic cell number, apoptosis incidence, and gene expression were analyzed with multiple regression, and those reflecting hatchability and karyotype were analyzed with logistic regression. The dichotomous-dependent variables (i.e., hatched or not hatched and diploid or non-diploid) were coded as 1 or 0. The analyzed independent variables are described in Table S2.

### Illustrating a logistic regression model for predicting the success or failure of pregnancy

OPU-IVF–derived embryos cultured in the microwell culture dishes were analyzed according to identified prognostic factors. Analyzed blastocysts were transferred into synchronized recipient cows (n = 52). After pregnancy diagnosis at day 30 after embryo transfer, the relationship between pregnancy status and prognostic factors was analyzed with logistic regression. The five prognostic factors and pregnancy status were converted to dummy variables. The dichotomous dependent variables (i.e., pregnant or not pregnant) were coded as 1 or 0, respectively. The predictive accuracy of the conventional selection method according to the International Embryo Transfer Society manual [1] was also analyzed with logistic regression [28]. The independent variables and its dummy variables are described in Table S3.

### Statistical analysis

All statistical analyses were performed with StatView version 5.0 (SAS Institute Inc., Cary, NC, USA), except for hierarchical cluster analysis, which was performed with JMP version 4.0.5 (SAS Institute Inc.), and receiver operating characteristic (ROC) plot analysis, which was performed by ROC plotting and area under the curve (AUC) calculation transferability test version 1.3–7 software [29].

## Results

### Optimization of Microwell Culture Dish Conditions

To maximize development to the blastocyst stage in *in vitro* culture with the mirowell culture dish, we optimized the oxygen tension (Table S4). We observed greater development to the blastocyst stage in 5% O_2_ as compared with 20% O_2_ (*P*<0.01). Thus, hypoxic conditions are suitable for microwell culture dish, and we used 5% O_2_ in subsequent studies.

### Tracking *In Vitro* Development of Individual Bovine Embryos with TLC Imaging and the Microwell Culture Dish

The 638 embryos that formed blastocysts reached the 2-, 3- or 4-, and 5- to 8-cell stage at 25.9±1.9, 35.2±2.5, and 43.2±3.2 hpi (mean ± SD), respectively. The lengths of the first, second, and third cell cycles were 25.9±1.9, 9.1±1.3, and 8.1±1.3 h, respectively. In the first cleavage, we observed some of embryos that underwent direct cleavage from a 1-cell blastomere to a 3- or 4-cell blastomere (Movie S1). Embryos subsequently underwent lag-phase either during the fourth cell cycle for 41.5±5.9 h or during the fifth cell cycle for 32.9±6.6 h following a short fourth cell cycle for 7.4±1.5 h (Movie S2). The lag-phase was observed in 99.6% of embryos that reached the blastocyst stage; however, the number of blastomeres at the onset of the phase varied from 4 to 16, and embryos were thus classified into three groups: 4–5, 6–8, and 9–16 blastomeres (Fig. S1). The end times of the phases were not different among embryos undergoing a long fourth cell cycle (83.0±7.8 hpi) and those undergoing a short fourth cell cycle (81.7±6.9 hpi). Embryos reached the morula, blastocyst, and expanded blastocyst stages at 101.0±7.7, 143.8±13.8, and 156.5±7.9 hpi, respectively.

### Oxygen Consumption at the Blastocyst Stage Is a Superior, Non-Invasive Predictor of Cell Numbers and Hatchability

The variables reflecting embryonic cell numbers were analyzed in 173 blastocysts with multiple regression analysis (Table S5). Oxygen consumption at the blastocyst stage was determined to be the best predictor of the number of total, ICM, and TE cells. The relationship between oxygen consumption and embryonic cell numbers including total, ICM, and TE cells showed a positive correlation, except for the percentage of ICM cells relative to total cell number (Fig. 2A–D). We furthermore found that the level of oxygen consumption was also a superior predictor of hatchability (Table S6). Blastocysts that later hatched had a higher oxygen consumption (1.12±0.37×10^−14^ mol s^−1^) as compared with blastocysts that did not hatch (0.80±0.26×10^−14^ mol s^−1^) (Fig. 2E). The rate of hatching among blastocysts increased with elevated oxygen consumption (Fig. 2F). For predicting the success or failure of hatching, the optimal cut-off point determined by the ROC curve (AUC 0.77; *P* = 0.03; 95% CI 0.705–0.837) was 0.84×10^−14^ mol s^−1^, which was the cut-off point used to differentiate between high and low oxygen consumption. The rate of hatching in blastocysts with high oxygen consumption (98/128, 76.6%) was more than double the rate in blastocysts with low oxygen consumption (23/67, 34.3%) (Fig. 2F). Overall success or failure of hatching was predicted 72.8% (142/195) of the time by the cut-off point, indicating that the competence of blastocysts to undergo subsequent development such as hatching could be accurately and objectively predicted by measuring oxygen consumption.

### A Small Number of Blastomeres at the Onset of the Lag-Phase Is Correlated with a Low ICM Percentage and High Apoptosis Incidence at the Blastocyst Stage

**Figure 3:**
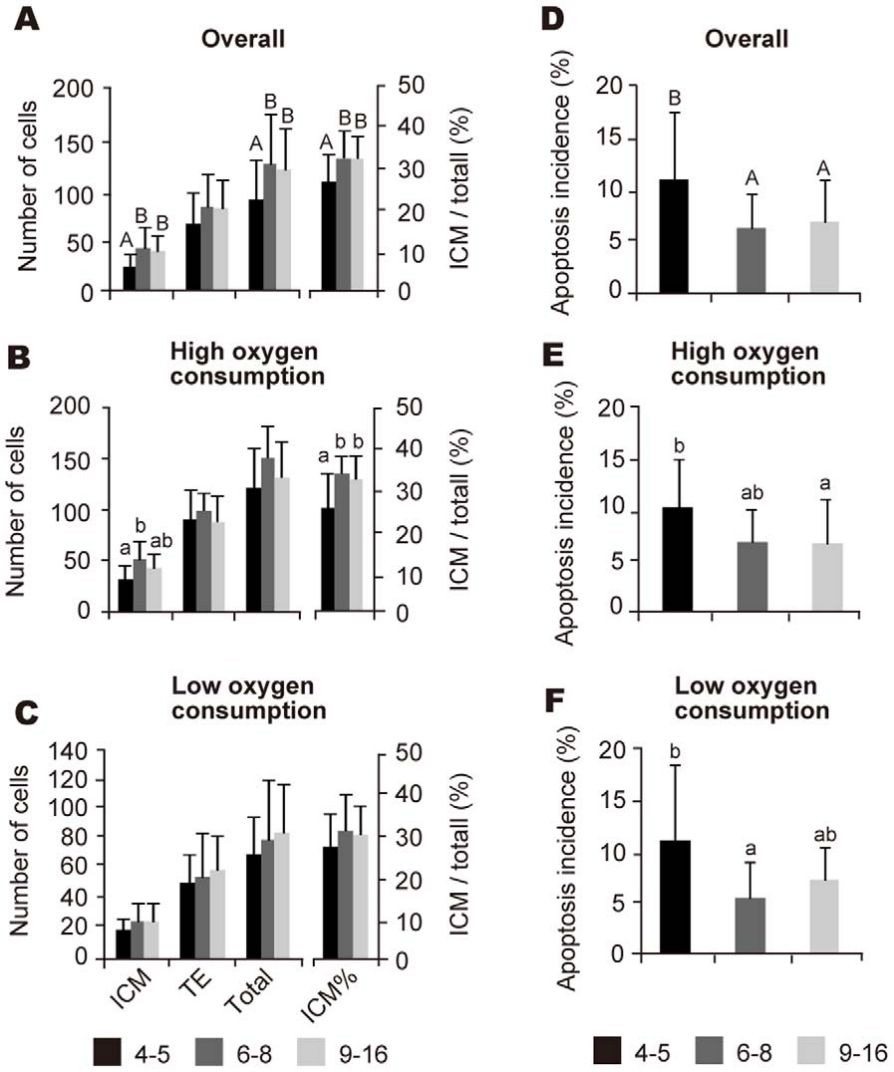
The effect of the number of blastomeres at the onset of the lag-phase on cell numbers, ICM ratio, and apoptosis incidence. The number of total, ICM, and TE cells, the ICM ratio, and the apoptotic incidence in blastocysts with different numbers of blastomeres at the onset of the lag-phase are shown in **A** and **D**, respectively. Embryos were separated into groups based on high (**B** and **E**) or low oxygen consumption (**C** and **D**) with a cut-off of 0.84×10^−14^ mol s^−1^. Data are presented as the mean ± SD. Different letters indicate significant differences between groups based on one-way ANOVA followed by Fisher's protected least signicicant difference (PLSD) (^A,B^ *P*<0.01; ^a,b^ *P*<0.05).

We searched for variables that reflected the ICM% and the apoptosis incidence and found that the number of blastomeres at the onset of the lag-phase was correlated with the ICM% and apoptosis incidence, but oxygen consumption was not (Tables S5 and S7). Overall, blastocysts with 4/5 blastomeres at the onset of the lag-phase showed a lower ICM% (26.4±7.5%) (Fig. 3A) and a higher incidence of apoptosis (measured as the percentage of cells undergoing apoptosis; 10.9±6.5%) (Fig. 3D) as compared with blastocysts with 6–8 blastomeres (ICM%, 32.4±6.8%; apoptosis incidence, 6.2±3.2%) and 9–16 blastomeres (ICM%, 32.0±6.2%; apoptosis incidence, 6.8±4.1%). In blastocysts with high oxygen consumption, embryos with 4/5 blastomeres at the onset of the lag-phase showed a lower ICM% (25.7±8.8%) (Fig. 3B) and a higher incidence of apoptosis (11.3±7.5%) (Fig. 3E) as compared with blastocysts with 6–8 blastomeres (ICM%, 33.5±5.5%; apoptosis incidence, 5.5±3.6%). Higher incidence of apoptosis in embryos with 4/5 blastomeres (11.3±7.5%) as compared with embryos with 6–8 blastomeres (5.5±3.6%) was also observed in blastocysts with lower oxygen consumption (Fig. 3F), but lower ICM% was not (Fig. 3C).

### Slow Cleaving of Blastocysts with 3/4 Blastomeres at the End of the First Cleavage May Indicate a High Incidence of Chromosomal Abnormality

**Figure 4:**
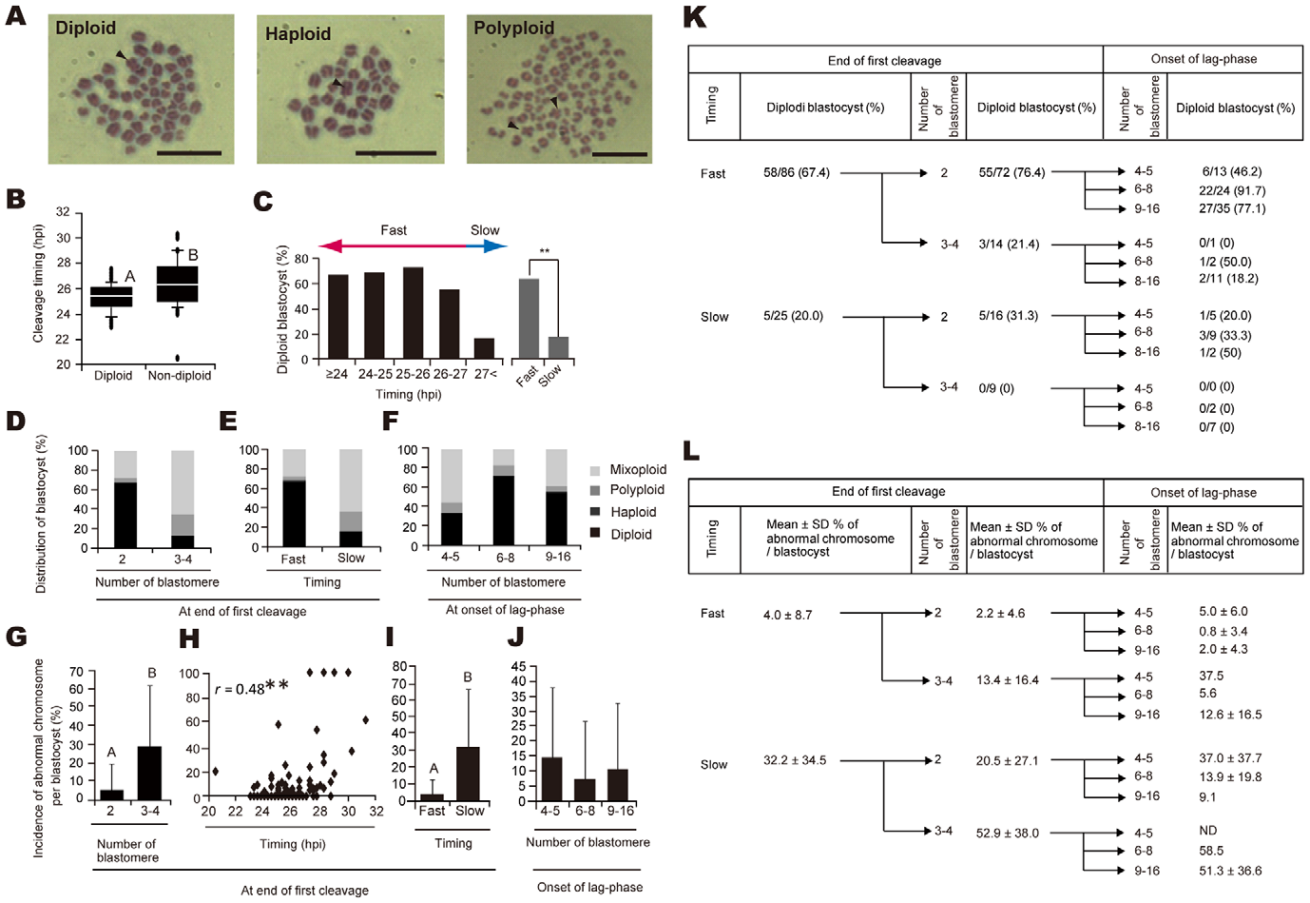
Involvement of cytokinesis during *in vitro* development on chromosome complementation. (**A**) Two sets (2n = 60), one set (n = 30), and more than two sets of chromosomes (3n, 4n, 5n etc.) were considered diploid, haploid, and polyploid, respectively. The arrowheads indicate the X chromosome. Bar = 10 µm. (**B**) The timing of the first cleavage in diploid embryos (defined as blastocysts in which all nuclei were diploid) and non-diploid embryos (defined as blastocysts with more or fewer than two sets of chromosomes in the analyzed nuclei). Different letters indicate significant differences between groups based on one-way ANOVA followed by Fisher's PLSD (^A,B^ *P*<0.01). (**C**) Relationship between timing of the first cleavage and the incidence of diploid embryos. Blastocysts were divided into two groups, fast and slow, based on the timing of the first cleavage with a cut-off of 27 hpi (**C**). Double Asterisks indicate significant differences between groups based on the χ^2^ test (*P*<0.01). Ploidy (**D**–**F**) and incidence of abnormal chromosomes per embryo (**G**–**J**) in blastocysts derived from embryos with 2 and 3/4 blastomeres at the end of the first cleavage (**D** and **G**); rapidly cleaving and slowly cleaving embryos at the end of the first cleavage (**E**, **H**, and **I**); and embryos with 4/5, 6–8, and 9–16 blastomeres at the onset of the lag-phase (**F** and **J**) are shown. **Coefficient of determination, *r*, was statistically significant based on simple regression analysis (*P*<0.01) (**H**). Different letters indicate significant differences between groups based on one-way ANOVA followed by Fisher's PLSD (^A,B^ *P*<0.01) (**I**). The incidence of diploid blastocysts (**K**) and the incidence of abnormal chromosomes per blastocyst (**L**) were classified according to the timing and blastomere number at the end of the first cleavage and the blastomere number at the onset of the lag-phase.

In 111 blastocysts, a total of 2187 mitotic cells were analyzed. The mitotic cells were analyzed and ranged from a minimum of 2 to a maximum of 47 mitotic cells per blastocyst. The mitotic index was 0.39±0.13 (mean ± SD). Variables reflecting the ploidy, cleavage timing, and number of blastomeres at the end of the first cleavage and the number of blastomeres at the onset of the lag-phase were correlated with diploidy of chromosomal complementation at the blastocyst stage (Table S8). Blastocysts identified as haploid, polyploid, or mixoploid displayed slower first cleavage (26.4±2.0 hpi) as compared with those with normal blastocysts identified as diploid (25.3±1.1 hpi) (*P*<0.05) (Fig. 4B). The incidence of diploid blastocysts decreased with a remarkably prolonged duration of the first cleavage (>27 hpi) (Fig. 4C). Based on this result, the timing of the first cleavage was considered a fast or slow cleavage event with a cut-off of 27 hpi (Fig. 4C). The majority of slowly cleaving embryos were mixoploid (67.4%), whereas the incidence of mixploidy in rapidly cleaving embryos was 27.9% (Fig. 4E). Furthermore, slowly cleaving embryos had a higher incidence of abnormal chromosomes per blastocyst compared with that of rapidly cleaving embryos (Fig. 4H). Blastocysts from embryos with 3/4 blastomeres at the end of the first cleavage (Fig. 4D) or with 4/5 blastomeres at the onset of the lag-phase (Fig. 4F) also had a higher rate of mixoploidy. Moreover, blastocysts derived from embryos with 3/4 blastomeres at the end of the first cleavage had higher incidences of abnormal chromosomes per blastocyst as compared with those with 2 blastomeres (Fig. 4G). Overall, diploid blastocysts were observed with a frequency of 56.8% (63/111). Most (77.8%; 49/63) blastocysts were derived from rapidly cleaving embryos with 2 blastomeres at the end of the first cleavage and either 6–8 or 9–16 blastomeres at the onset of the lag-phase. These blastocysts had a high possibility of diploidy (Fig. 4K) and a low incidence of abnormal chromosomes per blastocyst (Fig. 4L). We observed that accumulation of abnormal phenotypes at the first cleavage and at the onset of the lag-phase appeared to have an increasingly serious influence on chromosomal complementation (Fig. 4F and G).

### Aberrant Expression of *IFNΤ*, *IGF2R*, and *AKR1B1* in Blastocysts Undergoing an Abnormal First Cleavage

**Figure 5:**
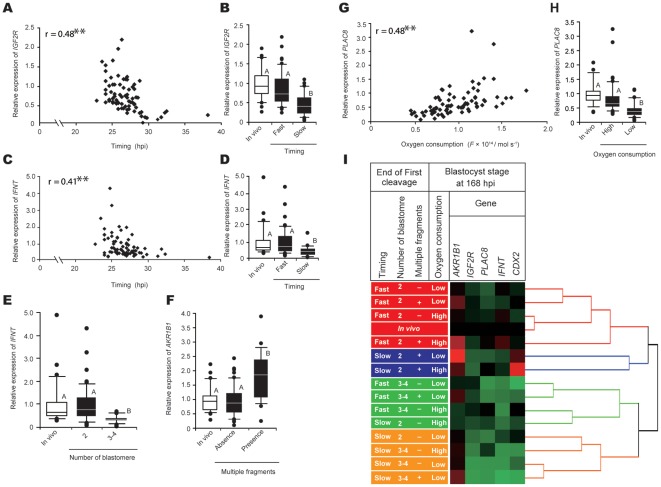
Factors relating to expression of *IGF2R*, *IFNΤ*, *AKR1B1*, and *PLAC8* at the blastocyst stage. Relationship between *IGF2R* and timing of the first cleavage (**A** and **B**), *IFNΤ* and timing of the first cleavage (**C** and **D**), *IFNΤ* and the number of blastomeres at the end of the first cleavage (**E**), *AKR1B1* and the presence or absence of multiple fragments at the end of the first cleavage (**F**), and *PLAC8* and oxygen consumption at the blastocyst stage (**G** and **H**) are shown. Relative expression is shown as the fold change from the mean value of *in vivo*–derived blastocysts, which was set at 1. **Coefficient of determination, *r*, was statistically significant based on simple regression analysis (*P*<0.01) (**A**, **C**, and **G**). Different letters indicate significant differences between groups based on one-way ANOVA followed by Fisher's PLSD (^A,B^ *P*<0.01) (**B**, **D**, **F**, and **H**). Gene expression in blastocysts classified based on timing, blastomere number, the presence or absence of multiple fragments at the end of the first cleavage, and oxygen consumption of blastocysts at 168 hpi (**I**). Embryos displaying similar patterns of expression for these five genes were grouped together on closely connected branches of the dendrogram with the same colors. The color map indicates normalized expression values that were based on *in vivo*–derived blastocysts for each gene examined. Red represents high expression; black, approximately equal expression; and green, low expression as compared with *in vivo*–derived blastocysts.

To identify factors correlated with the expression of genes that are reflective of high-quality embryos known to yield a pregnancy [8], variables related to five genes involved in pregnancy reorganization and placentation (*AKR1B1*, *IGF2R*, *PLAC8*, *IFNΤ*, and *CDX2*) were analyzed individually in 76 blastocysts (Table S9). We observed that the timing of the first cleavage was correlated with *IFN-tau* and *IGF2R* expression (Table S9). *IFNΤ* and *IGF2R* expression in embryos that cleaved at >27 hpi was lower than that in rapidly cleaving embryos and *in vivo*–derived embryos (Fig. 5A and B). *IFNΤ* expression was also related to the number of blastomeres at the end of the first cleavage (Table S9). *IFNΤ* expression in blastocysts with 3/4 blastomeres at the end of the first cleavage was lower as compared with those with 2 blastomeres and *in vivo*–derived embryos (Fig. 5C). *AKR1B1* expression was correlated with the presence or absence of multiple fragments (Table S9). The *AKR1B1* expression of embryos with multiple fragments at the end of the first cleavage was higher than the expression of embryos without fragments and those derived *in vivo* (Fig. 5D). Furthermore, oxygen consumption by blastocysts at 168 hpi was correlated with *PLAC8* (Table S6), and the expression level in blastocysts with low oxygen consumption was lower than that in blastocysts with high oxygen consumption and in blastocysts derived *in vivo* (Fig. 5E). As shown with the hierarchical cluster algorithm (Fig. 5F), four major groups were identified with gene expression profiles. Blastocysts derived from rapidly cleaving embryos with 2 blastomeres at the end of the first cleavage characterized the group derived *in vivo*. The gene expression profile in blastocysts with high oxygen consumption derived from rapidly cleaving embryos with 2 blastomeres and without fragmentation at the end of the first cleavage was closest to that of *in vivo*–derived blastocysts. In contrast, the expression profile of blastocysts derived from slowly cleaving embryos with 3/4 blastomeres at the end of the first cleavage appeared to be quite different from those derived *in vivo*. Expression of *IGF2R* and *IFNΤ* in that group was reduced to 30–40% as compared with expression in blastocysts derived *in vivo*.

### A Combination of Identified Prognostic Factors Largely Predicted the Success of Pregnancy

To assess whether the identified variables that were related to various markers of blastocyst quality could be functional prognostic factors, the relationship between pregnancy and the identified variables was analyzed by transferring 52 OPU-IVF embryos to 52 recipient cows. Logistic regression analysis showed that although no embryos that were assessed for individual identified variables or conventional variables could be used to predict pregnancy success, a combination of identified variables allowed the prediction of pregnancy success. Furthermore, the combination improved the overall percentage of blastocysts correctly classified as resulting in pregnancy or non-pregnancy (Table 1). The pregnancy rate of that time was 40.4% (21/52) and female rate was 57.1% (12/21). No significant difference was observed in timing of first cleavage (male, 25.3±1.4 hpi vs. female, 25.1±1.3 hpi) and oxygen consumption at blastocyst stage (male, 1.20±0.3×10^−14^ mol s^−1^ vs. female, 1.04±0.2×10^−14^ mol s^−1^) among sex in newborns. Using the full array of prognostic factors including timing of first cleavage, blastomere number at the end of first cleavage, presence or absence of multiple fragments at the end of the first cleavage, blastomere number at the onset of the lag-phase, and oxygen consumption at the blastocyst stage, 78.9% (15/19) of blastocysts predicted to result in pregnancy were correctly classified. The overall correct classification rate for predicting both classes of embryos was 80.8% (42/52), which was significantly higher than the conventional method (59.6%, 31/52) according to the stage and morphological code of blastocysts at the time of transfer (Table 1). Most (93.3%, 14/15) recipient cows that received blastocysts that satisfied all prognostic factors, namely, high oxygen consumption (≥0.84×10^−14^ mol s^−1^), fast cleavage (≤24.0 hpi), 2 blastomeres without multiple fragments at the end of the first cleavage, and 6–16 blastomeres at the onset of the lag-phase, delivered at 285–300 days. Percentage of female in the newborn was 60% (9/15). The birth weight of the newborns derived from embryos that satisfied all prognostic factors was 29.2±3.3 kg that was close to that derived from AI embryos (28.7±4.2 kg) (Fig. S2), and we observed no neonatal overgrowth or death.

**Table 1:** Classification matrices for selected regression models illustrating the prediction accuracy of variables examined either individually or in combination.

	Predicted outcome					
	Fate			Percentage correct		
Prognostic factor (PF) entered into the logistic model	Observed outcome	Not pregnant	Pregnant	Per fate class	Pregnant correctly classified^a^	Overall^b^
Embryo transfer: Stage and morphological code (conventional)^c^	Not pregnant	31	0	100	0	59.6^d^
	Pregnant	21	0	0		
First cleavage: Timing (PF1)	Not pregnant	31	0	100	0	59.6^d^
	Pregnant	21	0	0		
First cleavage: Number of blastomeres (PF2)	Not pregnant	31	0	100	0	59.6^d^
	Pregnant	21	0	0		
First cleavage: Presence or absence of multiple fragments (PF3)	Not pregnant	31	0	100	0	59.6^d^
	Pregnant	21	0	0		
Onset of lag-phase: Number of blastomeres (PF4)	Not pregnant	31	0	100	0	59.6^d^
	Pregnant	21	0	0		
Oxygen consumption of blastocysts at 168 hpi (PF5)	Not pregnant	31	0	100	0	59.6^d^
	Pregnant	21	0	0		
Combination of PF1 and PF2	Not pregnant	22	9	71.0	66.7	76.9^de^
	Pregnant	3	18	85.7		
Combination of PF1, PF2, and PF3	Not pregnant	24	7	77.4	70.8	78.8^de^
	Pregnant	4	17	81.0		
Combination of PF1, PF2, PF3, and PF4	Not pregnant	25	6	80.6	72.7	78.8^de^
	Pregnant	5	16	76.2		
Combination of PF1, PF2, PF3, PF4, and PF5	Not pregnant	27	4	87.1	78.9	80.8^e^
	Pregnant	6	15	71.4		

a The percentage of pregnancies correctly classified as a proportion of those blastocysts predicted to result in a successful pregnancy.

b The percentage of embryos correctly classified as resulting in no pregnancy and pregnancy (the hit ratio).

c Conventional variables based on the stage and morphological quality code of blastocysts at the time of transfer.

d,e Different letters indicate a significant difference (P<0.05). Data were analyzed with the Yates' corrected χ^2^ test.

## Discussion

We have established a novel system for selecting bovine embryos for transfer using a combination of TLC imaging with microwell culture dish and oxygen consumption analysis by SECM. This system was successful in identifying five prognostic factors: (i) timing of the first cleavage, (ii) blastomere number at the end of the first cleavage, (iii) presence or absence of multiple fragments at the end of the first cleavage, (iv) blastomere number at the onset of the lag-phase, and (v) oxygen consumption at the blastocyst stage. These factors not only reflected blastocyst quality but also viability after transfer.

The timing of certain events during the earliest stage of development may be linked to subsequent embryonic viability [30]. In particular, the relationship between the timing of the initial division and embryonic viability has shown that slowly cleaving embryos have a lower likelihood of pregnancy than rapidly cleaving embryos [31]. The reasons why slowly cleaving embryos have inferior viability and lead to lower pregnancy and implantation rates are unknown; however, chromosomal aberration may play a role and likely reflects the quality of spermatozoa and oocytes [32]. We confirmed here that slowly cleaving embryos had a higher incidence of abnormal chromosomes and were identified as mixoploid more often than rapidly cleaving embryos. A similar correlation was also found in blastocysts derived from embryos with 3/4 blastomeres at the end of the first cleavage or from those with 4/5 blastomeres at the onset of the lag-phase. Because mixoploidy occurs in a relatively large number of *in vitro*–produced embryos, mixoploidy does not seem to critically influence subsequent development. A high frequency of abnormal chromosomes per blastocyst (>25%) may, however, have serious consequences [33].

Along with abnormal chromosomes, we observed downregulation of *IFNΤ* expression in slowly cleaving embryos and embryos with 3/4 blastomeres at the end of the first cleavage. The *IFNΤ* gene is regulated by *ETS-2* and is believed to be involved in the establishment of pregnancy and signaling of maternal recognition of pregnancy in ruminants [34]–[36]. A balance of expression seems to be disrupted by aberrant epigenetic regulation because *IFNΤ* mRNA can be regulated by high *H3K18* acetylation and low *H3K9* methylation [37]. An important relationship between *IFNΤ* expression and pregnancy is unproven, but low expression of *IFNΤ* may lead to serious placental abnormalities [38], [39]. Cytogenetic and epigenetic errors that were identified by chromosome abnormalities and downregulation of *IFNΤ* expression may therefore be concomitantly or autonomously involved in the subsequent development of embryos with problematic phenotypes during the early cleavage stage.

Fragmentation, which was identified as a prognostic factor, is a morphologic feature that has been recognized as a determinant of viability and is likely to produce insufficient oocyte maturation, which compromises cytoskeletal patency. Fragmentation may distort blastomere division planes, leading to abnormal compaction, cavitation, and blastocyst formation [40]. The spatial relationships may be restored with fragment removal, leading to improved blastulation, implantation, and live-birth rates [40]. In our current study, we observed upregulation of *AKR1B1* in blastocysts derived from embryos with multiple fragments at the end of the first cleavage. *AKR1B1* encodes aldolase reductase, an enzyme that is involved in both progesterone metabolism and prostaglandin F2α synthesis, which subsequently terminates the pregnancy. Upregulation of *AKR1B1* has been associated with embryos that fail to establish pregnancies or that are resorbed [8]. Upregulation of aldose reductase is induced by external stimulation such as oxidative and osmotic stress [41], [42]. The mechanism of how multiple fragments at the end of the first cleavage affect the expression of *AKR1B1* is unclear. This may be a secondary phenomenon following spatial stress from direct or indirect effects of multiple fragments that are present at the end of the first cleavage.

TLC imaging showed that a low number of blastomeres at the onset of the lag-phase was related to a low ICM% and elevated apoptosis in blastocysts. The lag-phase during the fourth or fifth cell cycle, which corresponds to the 5- to 8-cell stage and to the 9- to 16-cell stage, respectively, is a key step in embryonic gene activation (EGA), which occurs after the maternal-to-zygotic transition [43]. Moreover, the fourth cell cycle may be a conserved step in cell differentiation into ICM or TE cells. Indeed, the first signs of cell polarity in bovine embryos can already be detected at that stage [44]. Parameters of the lag-phase may, therefore, reflect differentiation and/or EGA and may consequently be related to cell allocation and apoptosis at the blastocyst stage.

One additional potential predictor of embryonic viability included in our system was oxygen consumption at the blastocyst stage. Previously, we determined that anomalous oxygen consumption in porcine embryos at the blastocyst stage could indicate reduced hatchability [19]. In our current study, we found that decreased oxygen consumption reflected a low cell number and reduced hatchability, which may be induced by inferior cell growth in bovine embryos (Fig. S3). Similarly, decreased oxygen consumption may be a sign of aberrant mitochondrial function and metabolism, which may result in compromised fetal and placental outcomes [45]. This possibility is implied by the finding that blastocysts with low oxygen consumption showed downregulation of *PLAC8*, which may be a marker gene for embryo apposition and pregnancy induction [8]. In fact, the mean level of oxygen consumption in successful pregnancies (1.11±0.23×10^−14^ mol s^−1^) was higher than that in failed pregnancies (0.95±0.27×10^−14^ mol s^−1^) (Fig. S4).

The ability to predict embryo viability after transfer is an important goal for stable and efficient production of healthy newborns. Although establishment of a prognostic model appears to be necessary to accomplish this goal in cattle, limited information about predictors that are related to embryo viability after transfer is available, and no system has been established. We therefore examined the practical advantage of the identified factors in predicting post-transfer viability of OPU-IVF embryos. Pregnancy outcome was confirmed with a logistic regression model. Although individual identified variables and conventional variables based on morphological quality at the time of embryo transfer were not useful in defining pregnancy success, the multiple predictors we identified, including mitotic events at the end of the first cleavage and at the lag-phase as well as oxygen consumption, enabled us to predict pregnancy success and improved the success rate of the predictions. Our results suggest that the prediction of pregnancy success or failure may be nearly impossible with the conventional method or with individual factors reflecting blastocyst quality. Although we observed pregnancy in a few recipient cows that received blastocysts with an abnormal phenotype as detected with TLC analysis and measurement of oxygen consumption, the chance of pregnancy appeared to be progressively decreased with an increasing number of abnormal phenotypes (Table S10). This result is supported by previous study showing that individual predictor such as timing of first cleavage is not crucial for pregnancy success in cattle [16]. It may therefore not be possible to identify a crucial factor that determines embryo viability.

Neonatal and postnatal phenotypes may be altered by embryo production *in vitro*; however, the trigger and mechanism are unknown. In our present study, we observed no neonatal death and no overgrowth such as large offspring syndrome (LOS) in our newborn calves. Fetal growth and neonatal phenotypes appear to be particularly sensitive to an imbalance in imprinting gene expression. Young et al. suggested that LOS may be associated with an epigenetic change in *IGF2R*, resulting in reduced expression of *IGF2R* [46]. In our present study, reduction of *IGF2R* expression was observed in blastocysts derived from slowly cleaving embryos. We hypothesize that slow first cleavage is implicated in neonatal phenotypes such as LOS. Hence, selection of blastocysts with multiple predictors may allow prediction not only of the success or failure of pregnancy but also of neonatal health. Examining the possibility of a direct causal relationship between developmental kinetics *in vitro* and neonatal phenotypes will be an interesting future subject. In conclusion, our proposed novel system and multiple predictors could be used for practical selection of healthy IVF bovine embryos as an alternative to conventional morphological assessment at the time of transfer.

## Supporting Information

Figure S1 **Timing of the appearance of lage-phase and number of blastomere at 61 hpi.** Percentage of embryos that began the lag-phase (**A**). From TLC analysis, 100% of embryos that reached the blastocyst stage (n = 97) began lag-phase between 56.0 and 67.0 hpi (black line). The incidence of embryos observed in lag-phase in the fourth (red line) and fifth cell cycle (blue line) reached a plateau at 52.0 and 56.0 hpi, respectively. This plateau was maintained until 67.0 hpi. According to this observation, the number of blastomeres was counted at 61 hpi when all embryos appeared to be in lag-phase (**B**). To count the actual number of blastomeres at this time, the zona pellucida was removed by treating the embryos with 0.25% pronase (Kaken Pharmaceutical Co. Ltd., Tokyo, Japan) in Dulbecco's phosphate-buffered saline (DPBS; GIBCO BRL) for 1–2 min, followed by gentle pipetting with a tapered Pasteur pipette to separate the blastomeres. At 61 hpi, the most frequent numbers of blastomeres were five, eight, and nine. Based on this result, the number of blastomeres was categorized into three groups: 4/5, 6–8, and 9–16 blastomeres.
(EPS)

Figure S2 **The birth weight of offspring derived from artificial inseminated (AI) embryos,** ***in vitro*** **- fertilized (IVF) embryos that satisfied all prognostic factors: embryos accomplished the first cleavage within 27 hpi without fragmentation at the end of the first cleavage, 2 blastomere at end of first cleavage, >5 blastomeres at the onset of the lag-phase, and ≥0.84×10^−14^ mol s^−1^ oxygen consumption at the blastocyst stage (IVF1), and embryos that selected by conventional method: embryos was code 1 and expanded blastocyst stage (IVF2).** The boxes indicate the 25th and 75th percentiles, and the middle horizontal line indicates the median. Whiskers indicate the maximum and minimum values within the acceptable range defined by the two quartiles. Circles denote outliers. Data were analyzed with one-way ANOVA and Fisher's PLSD.
(EPS)

Figure S3 **Correlation between oxygen consumption and mitotic index in blastocysts.** The mitotic index is well recognized as an indicator of competence for cell growth. The correlation coefficient was determined with simple regression analysis. The coefficient of determination, *r*, was statistically significant at *P*<0.01.
(EPS)

Figure S4 **Effect of oxygen consumption at blastocyst stage on pregnancy success.** The boxes indicate the 25th and 75th percentiles, and the middle horizontal line indicates the median. Whiskers indicate the maximum and minimum values within the acceptable range defined by the two quartiles. Circles denote outliers. Data were analyzed with the Student *t*-test. Different letters are significantly difference at ^a,b^ *P*<0.05.
(EPS)

Movie S1 **Time-lapse cinematography imaging of IVF bovine embryos cleaved from one cell to two blastomeres (A) and directly cleaved from one cell to four blastomeres (B).**
(MOV)

Movie S2 **Time-lapse cinematography imaging in IVF bovine embryos that underwent long (A) and short fourth cell cycles (B).**
(MOV)

Table S1 **Sequences of primers used for real-time RT-PCR.**
(DOC)

Table S2 **The list of variables examined in current study.**
(DOC)

Table S3 **Identified variables reflecting blastocysts qualities and conventional variables according to International Embryo Transfer Society manual.**
(DOC)

Table S4 **Effect of oxygen tension on** ***in vitro*** **development of bovine IVF embryos cultured in microwell culture dish.**
(DOC)

Table S5 **Multiple regression analysis of variables from blastocysts (n = 173) reflecting cell number and cell allocation.**
(DOC)

Table S6 **Logistic regression analysis of variables from blastocysts (n = 195) reflecting hatchability.**
(DOC)

Table S7 **Multiple regression analysis of variables from blastocysts (n = 74) reflecting apoptosis incidence.**
(DOC)

Table S8 **Logistic regression analysis of variables from blastocysts (n = 111) reflecting the karyotype.**
(DOC)

Table S9 **Multiple regression analysis of variables from blastocysts (n = 75) reflecting gene expression.**
(DOC)

Table S10 **Estimated probabilities derived from the fitted logistic regression model of pregnancy success.**
(DOC)
