# Supplementary material for: Promising System for Selecting Healthy In Vitro–Fertilized Embryos in Cattle
Source: PLoS One. 2012 May 9;7(5):e36627. doi: 10.1371/journal.pone.0036627 (PMC3348877; doi:10.1371/journal.pone.0036627)
Supplement: Table S1 — Sequences of primers used for real-time RT-PCR. (DOC) [file pone.0036627.s007.doc]

Table S1

| Gene name | GenBank accession no. | Sequences (5′–3′) | Amplicon size (bp) |
| --- | --- | --- | --- |
| *CDX2* | XM_871005 | F: GCCACCATGTACGTGAGCTAC | 140 |
|  |  | R: ACATGGTATCCGCCGTAGTC |  |
| *IFNΤ* | X65539 | F: TCCATGAGATGCTCCAGCAGT | 103 |
|  |  | R: TGTTGGAGCCCAGTGCAGA |  |
| *IGF2R* | NM_174352.2 | F: GCTGCGGTGTGCCAAGTGAAAAAG | 201 |
|  |  | R: AGCCCCTCTGCCATTGTTACCT |  |
| *PLAC8* | NM_016619 | F: CGGTGTTCCAGAGGTTTTTCC | 163 |
|  |  | R: AAGATGCCAGTCTGCCAGTCA |  |
| *AKR1B1* | NM_001012519.1 | F: CGTGATCCCCAAGTCAGTGA | 152 |
|  |  | R: AATCCCTGTGGGAGGCACA |  |
| *H2AFZ* | NM_174809 | F: ACAGCTGTCCAGTGTTGGTG | 125 |
|  |  | R: GCAGAAATTTGGTTGGTTGG |  |
