# Supplementary material for: Promising System for Selecting Healthy In Vitro–Fertilized Embryos in Cattle
Source: PLoS One. 2012 May 9;7(5):e36627. doi: 10.1371/journal.pone.0036627 (PMC3348877; doi:10.1371/journal.pone.0036627)
Supplement: Table S2 — The list of variables examined in current study. (DOC) [file pone.0036627.s008.doc]

Table S2

| First cleavage: Timing | Continuous variable |
| --- | --- |
| First cleavage: Number of blastomeres | 2 blastomeres (1) |
|  | 3/4 blastomeres (0) |
| First cleavage: Unevenness division^a^ | Unevenness (1) |
|  | Evenness (0) |
| First cleavage: Presence or absence of multiple fragments^b^ | Presence (1) |
|  | Absence (0) |
| Second cell cycle: Duration | Continuous variable |
| Third cell cycle: Duration | Continuous variable |
| Cell cycle observed lag-phase | Fourth cycle (1) |
|  | Fifth cycle (0) |
| Lag-phase: Duration | Continuous variable |
| Onset of lag-phase: Number of blastomeres | 4/5 blastomeres (1, 0) |
|  | 6-8 blastomeres (0, 1) |
|  | 9-16 blastomeres (0, 0) |
| Onset of lag-phase: Unevenness division | Unevenness (1) |
|  | Evenness (0) |
| Onset of lag-phase: Presence or absence of multiple fragments | Presence (1) |
|  | Absence (0) |
| Blastocyst at 168 hpi: Oxygen consumption | Continuous variable |

Dummy variables were indicated within parenthesis.

^a^ Uneven was defined as embryos in which there was more than a 20% difference in size/volume between the blastomeres (1).

^b^ Multiple fragments was defined as embryos with ≥10% fragmentation (2).

**References**

1. Scott L, Finn A, O'Leary T, McLellan S, Hill J. [Morphologic parameters of early cleavage-stage embryos that correlate with fetal development and delivery: prospective and applied data for increased pregnancy rates.](http://www.ncbi.nlm.nih.gov/pubmed/16982662) Hum Reprod 22: 230-240.

2. Ziebe S, Petersen K, Lindenberg S, Andersen AG, Gabrielsen A, et al. (1997) [Embryo morphology or cleavage stage: how to select the best embryos for transfer after in-vitro fertilization.](http://www.ncbi.nlm.nih.gov/pubmed/9262293) Hum Reprod 12: 1545-1549.
