# Supplementary material for: Promising System for Selecting Healthy In Vitro–Fertilized Embryos in Cattle
Source: PLoS One. 2012 May 9;7(5):e36627. doi: 10.1371/journal.pone.0036627 (PMC3348877; doi:10.1371/journal.pone.0036627)
Supplement: Table S3 — Identified variables reflecting blastocysts qualities and conventional variables according to International Embryo Transfer Society manual. (DOC) [file pone.0036627.s009.doc]

Table S3.

| Identified variables | First cleavage: Timing | Fast: ≤ 27.0 hpi (1) |
| --- | --- | --- |
|  |  | Slow: > 27.0 hpi (0) |
|  | First cleavage: Number of blastomeres | 2 blastomeres (1) |
|  |  | 3/4 blastomeres (0) |
|  | First cleavage: Presence or absence of multiple fragments | Presence (1) |
|  |  | Absence (0) |
|  | Onset of lag-phase: Number of blastomeres | 6-16 blastomeres (1) |
|  |  | 4/5 blastomeres (0) |
|  | Blastocyst at 168 hpi: Oxygen consumption | High: ≥ 0.84 × 10^–14^ mol s^–1^ (1) |
|  |  | Low: < 0.84 × 10^–14^ mol s^–1^ (0) |
| Conventional variables | Blastocysts at the time of transfer: Stage | Expanded blastocyst (1) |
|  |  | Blastocyst (0) |
|  | Blastocysts at the time of transfer: Morphological quality code | Code 1 (1, 0) |
|  |  | Code 2 (0, 1) |
|  |  | Code 3 (0, 0) |

Dummy variables were indicated within parenthesis.
