# Supplementary material for: Promising System for Selecting Healthy In Vitro–Fertilized Embryos in Cattle
Source: PLoS One. 2012 May 9;7(5):e36627. doi: 10.1371/journal.pone.0036627 (PMC3348877; doi:10.1371/journal.pone.0036627)
Supplement: Table S4 — Effect of oxygen tension on in vitro development of bovine IVF embryos cultured in microwell culture dish. (DOC) [file pone.0036627.s010.doc]

Table S4

| Oxygen tensions | No. of cultured embryos (replicates) | Percentages of embryos (mean ± SD%) | | | | |
| --- | --- | --- | --- | --- | --- | --- |
|  |  | Cleaved | |  | Developed to blastocyst stage | |
|  |  | Total | 4 cell < |  | Total | Expanded stage |
| 20% | 196 (9) | 83.6 ± 9.9 | 60.1 ± 12.9 |  | 39.0 ± 10.9^a^ | 28.0 ± 10.4 |
| 5% | 225 (9) | 86.7 ± 9.8 | 63.6 ± 16.2 |  | 56.0 ± 9.6^b^ | 35.3 ± 15.3 |

^a-b^ Different letter indicate significant difference (*P* < 0.01). Data that was arcsine transferred was analyzed by using Student t-test.
