# Supplementary material for: Promising System for Selecting Healthy In Vitro–Fertilized Embryos in Cattle
Source: PLoS One. 2012 May 9;7(5):e36627. doi: 10.1371/journal.pone.0036627 (PMC3348877; doi:10.1371/journal.pone.0036627)
Supplement: Table S5 — Multiple regression analysis of variables from blastocysts (n = 173) reflecting cell number and cell allocation. (DOC) [file pone.0036627.s011.doc]

Table S5

| Dependent variable | Variables | β^a^ | SEM^b^ | *t*−value | *P*−value | 95% C.I.^c^ |
| --- | --- | --- | --- | --- | --- | --- |
| Number of ICM cells | First cleavage: Timing | -0.964 | 0.534 | -1.805 | 0.073 | -2.020 to 0.091 |
|  | First cleavage: 2 blastomeres | 2.851 | 2.616 | 1.090 | 0.277 | -2.315 to 8.018 |
|  | First cleavage: Unevenness of division | -4.190 | 2.591 | -1.617 | 0.108 | -9.306 to 0.927 |
|  | First cleavage: Presence of multiple fragments | -2.315 | 2.652 | -0.873 | 0.384 | -7.553 to 2.922 |
|  | Second cell cycle: Duration | -1.482 | 1.084 | -1.368 | 0.173 | -3.622 to 0.658 |
|  | Third cell cycle: Duration | -0.220 | 0.914 | -0.241 | 0.810 | -2.026 to1.585 |
|  | Cell cycle observed at lag-phase | -0.181 | 3.594 | -0.050 | 0.960 | -7.280 to 6.918 |
|  | Lag-phase: Duration | 0.029 | 0.137 | 0.214 | 0.831 | -0.242 to 0.301 |
|  | Onset of lag-phase: 4/5 blastomeres | -10.295 | 3.776 | -2.726 | 0.007 | -17.753 to -2.836 |
|  | Onset of lag-phase: 6-8 blastomeres | 2.060 | 3.443 | 0.598 | 0.550 | -4.739 to 8.859 |
|  | Onset of lag-phase: Unevenness of division | 1.630 | 2.214 | 0.736 | 0.463 | -2.742 to 6.002 |
|  | Onset of lag-phase: Presence of multiple fragments | 3.996 | 2.668 | 1.498 | 0.136 | -1.273 to 9.265 |
|  | Blastocysts at 168 hpi: Oxygen consumption | 12.830 | 2.416 | 5.311 | 0.000 | 8.059 to 17.602 |
| Number of TE cells | First cleavage: Timing | -1.528 | 1.144 | -1.336 | 0.184 | -3.787 to 0.731 |
|  | First cleavage: 2 blastomeres | 8.871 | 5.599 | 1.584 | 0.115 | -2.187 to 19.930 |
|  | First cleavage: Unevenness of division | -7.791 | 5.545 | -1.405 | 0.162 | -18.743 to 3.161 |
|  | First cleavage: Presence of multiple fragments | -8.196 | 5.676 | -1.444 | 0.151 | -19.407 to 3.015 |
|  | Second cell cycle: Duration | 0.285 | 2.319 | 0.123 | 0.902 | -4.296 to 4.865 |
|  | Third cell cycle: Duration | -1.048 | 1.957 | -0.536 | 0.593 | -4.913 to 2.817 |
|  | Cell cycle observed at lag-phase | -4.104 | 7.694 | -0.533 | 0.595 | -19.298 to 11.091 |
|  | Lag-phase: Duration | -0.213 | 0.294 | -0.723 | 0.471 | -0.794 to 0.368 |
|  | Onset of lag-phase: 4/5 blastomeres | 0.247 | 8.083 | 0.031 | 0.976 | -15.717 to 16.211 |
|  | Onset of lag-phase: 6-8 blastomeres | 0.574 | 7.369 | 0.078 | 0.938 | -13.979 to 15.126 |
|  | Onset of lag-phase: Unevenness of division | -3.354 | 4.738 | -0.708 | 0.480 | -12.712 to 6.003 |
|  | Onset of lag-phase: Presence of multiple fragments | 2.310 | 5.710 | 0.405 | 0.686 | -8.967 to 13.587 |
|  | Blastocysts at 168 hpi: Oxygen consumption | 26.829 | 5.171 | 5.189 | 0.000 | 16.616 to 37.041 |
| Number of total cells | First cleavage: Timing | -2.492 | 1.502 | -1.659 | 0.099 | -5.459 to 0.475 |
|  | First cleavage: 2 blastomeres | 11.722 | 7.354 | 1.594 | 0.113 | -2.803 to 26.247 |
|  | First cleavage: Unevenness of division | -11.980 | 7.284 | -1.645 | 0.102 | -26.365 to 2.405 |
|  | First cleavage: Presence of multiple fragments | -10.511 | 7.456 | -1.410 | 0.161 | -25.236 to 4.213 |
|  | Second cell cycle: Duration | -1.198 | 3.046 | -0.393 | 0.695 | -7.214 to 4.819 |
|  | Third cell cycle: Duration | -1.268 | 2.570 | -0.493 | 0.622 | -6.344 to 3.808 |
|  | Cell cycle observed at lag-phase | -4.285 | 10.105 | -0.424 | 0.672 | -24.243 to 15.673 |
|  | Lag-phase: Duration | -0.183 | 0.386 | -0.474 | 0.636 | -0.946 to 0.580 |
|  | Onset of lag-phase: 4/5 blastomeres | -10.048 | 10.617 | -0.946 | 0.345 | -31.016 to 10.920 |
|  | Onset of lag-phase: 6-8 blastomeres | 2.634 | 9.678 | 0.272 | 0.786 | -16.481 to 21.748 |
|  | Onset of lag-phase: Unevenness of division | -1.724 | 6.223 | -0.277 | 0.782 | -14.015 to 10.567 |
|  | Onset of lag-phase: Presence of multiple fragments | 6.306 | 7.500 | 0.841 | 0.402 | -8.506 to 21.118 |
|  | Blastocysts at 168 hpi: Oxygen consumption | 39.659 | 6.792 | 5.839 | 0.000 | 26.246 to 53.072 |
| ICM% | First cleavage: Timing | 0.022 | 0.322 | 0.069 | 0.945 | -0.613 to 0.658 |
|  | First cleavage: 2 blastomeres | -2.483 | 1.576 | -1.576 | 0.117 | -5.595 to 0.629 |
|  | First cleavage: Unevenness of division | -0.077 | 1.561 | -0.049 | 0.961 | -3.159 to 3.005 |
|  | First cleavage: Presence of multiple fragments | 0.540 | 1.597 | 0.338 | 0.736 | -2.615 to 3.695 |
|  | Second cell cycle: Duration | -1.267 | 0.653 | -1.941 | 0.054 | -2.556 to 0.022 |
|  | Third cell cycle: Duration | 0.171 | 0.551 | 0.310 | 0.757 | -0.917 to1.258 |
|  | Cell cycle observed at lag-phase | -1.507 | 2.165 | -0.696 | 0.487 | -5.783 to2.769 |
|  | Lag-phase: Duration | 0.083 | 0.083 | 1.008 | 0.315 | -0.080 to 0.247 |
|  | Onset of lag-phase: 4/5 blastomeres | -4.958 | 2.275 | -2.180 | 0.031 | -9.451 to -0.465 |
|  | Onset of lag-phase: 6-8 blastomeres | 3.336 | 2.074 | 1.609 | 0.110 | -0.759 to 7.432 |
|  | Onset of lag-phase: Unevenness of division | 1.666 | 1.333 | 1.250 | 0.213 | -0.967 to 4.300 |
|  | Onset of lag-phase: Presence of multiple fragments | 1.600 | 1.607 | 0.996 | 0.321 | -1.574 to 4.774 |
|  | Blastocysts at 168 hpi: Oxygen consumption | 0.162 | 1.455 | 0.111 | 0.912 | -2.712 to 3.036 |

^a^ Coefficient estimate of multiple regression.

^b^ Standard error of β.

^c^ 95% confidence interval.
