# Supplementary material for: Promising System for Selecting Healthy In Vitro–Fertilized Embryos in Cattle
Source: PLoS One. 2012 May 9;7(5):e36627. doi: 10.1371/journal.pone.0036627 (PMC3348877; doi:10.1371/journal.pone.0036627)
Supplement: Table S6 — Logistic regression analysis of variables from blastocysts (n = 195) reflecting hatchability. (DOC) [file pone.0036627.s012.doc]

Table S6

| Variables | β^a^ | SEM^b^ | χ2 | *P*-value^c^ | Odds ratio | 95% C.I.^d^ |
| --- | --- | --- | --- | --- | --- | --- |
| First cleavage: Timing | -0.075 | 0.118 | 0.401 | 0.527 | 0.928 | 0.736 to 1.170 |
| First cleavage: 2 blastomeres | 2.21 | 1.348 | 2.687 | 0.101 | 9.112 | 0.649 to 128.013 |
| First cleavage: Unevenness of division | 0.536 | 0.514 | 1.088 | 0.297 | 1.71 | 0.624 to 4.684 |
| First cleavage: Presence of multiple fragments | -0.909 | 0.54 | 2.83 | 0.093 | 0.403 | 0.140 to 1.162 |
| Second cell cycle: Duration | 0.235 | 0.233 | 1.015 | 0.314 | 1.264 | 0.801 to 1.996 |
| Third cell cycle: Duration | 0.16 | 0.202 | 0.628 | 0.428 | 1.174 | 0.790 to 1.744 |
| Cell cycle observed at lag-phase | -0.019 | 0.032 | 0.328 | 0.567 | 0.982 | 0.921 to 1.046 |
| Lag-phase: Duration | 1.243 | 1.437 | 0.748 | 0.387 | 3.466 | 0.207 to 57.992 |
| Onset of lag-phase: 4/5 blastomeres | -1.806 | 1.371 | 1.734 | 0.188 | 0.164 | 0.011 to 2.416 |
| Onset of lag-phase: 6-8 blastomeres | -1.311 | 1.403 | 0.873 | 0.35 | 0.27 | 0.017 to 4.216 |
| Onset of lag-phase: Unevenness of division | 0.382 | 0.536 | 0.511 | 0.475 | 1.467 | 0.513 to 4.193 |
| Onset of lag-phase: Presence of multiple fragments | 0.327 | 0.599 | 0.299 | 0.585 | 1.387 | 0.429 to 4.488 |
| Blastocysts at 168 hpi: Oxygen consumption | 3.707 | 0.695 | 28.418 | <0.001 | 40.721 | 10.421 to 159.129 |

^a^ Coefficient estimate of multiple regression.

^b^ Standard error of β.

^c^ *P*-value of chi-square (χ^2^) statistic.

^d^ 95% confidence interval.
