# Supplementary material for: Promising System for Selecting Healthy In Vitro–Fertilized Embryos in Cattle
Source: PLoS One. 2012 May 9;7(5):e36627. doi: 10.1371/journal.pone.0036627 (PMC3348877; doi:10.1371/journal.pone.0036627)
Supplement: Table S7 — Multiple regression analysis of variables from blastocysts (n = 74) reflecting apoptosis incidence. (DOC) [file pone.0036627.s013.doc]

Table S7

| Variables | β^a^ | SEM^b^ | *t*-value | *P*-value | 95% C.I.^c^ |
| --- | --- | --- | --- | --- | --- |
| First cleavage: Timing | -0.220 | 0.220 | -1.003 | 0.319 | -0.658 to 0.217 |
| First cleavage: 2 blastomeres | -0.858 | 2.011 | -0.427 | 0.671 | -4.860 to 3.144 |
| First cleavage: Unevenness of division | -0.957 | 1.098 | -0.871 | 0.386 | -3.142 to 1.228 |
| First cleavage: Presence of multiple fragments | -0.549 | 1.254 | -0.438 | 0.663 | -3.045 to 1.946 |
| Second cell cycle: Duration | -0.385 | 0.351 | -1.097 | 0.276 | -1.085 to 0.314 |
| Third cell cycle: Duration | -0.518 | 0.391 | -1.325 | 0.189 | -1.296 to 0.260 |
| Cell cycle observed at lag-phase | 1.092 | 2.158 | 0.506 | 0.614 | -3.202 to 5.386 |
| Lag-phase: Duration | -0.024 | 0.078 | -0.304 | 0.762 | -0.178 to 0.131 |
| Onset of lag-phase: 4/5 blastomeres | 5.713 | 2.282 | 2.504 | 0.014 | 1.173 to 10.254 |
| Onset of lag-phase: 6-8 blastomeres | -0.187 | 2.329 | -0.080 | 0.936 | -4.822 to 4.447 |
| Onset of lag-phase: Unevenness of division | -1.007 | 1.271 | -0.792 | 0.431 | -3.535 to 1.522 |
| Onset of lag-phase: Presence of multiple fragments | -2.202 | 1.298 | -1.696 | 0.094 | -4.785 to 0.381 |
| Blastocysts at 168 hpi: Oxygen consumption | -1.298 | 1.431 | -0.907 | 0.367 | -4.146 to 1.550 |

^a^ Coefficient estimate of multiple regression.

^b^ Standard error of β.

^c^ 95% confidence interval.
