# Supplementary material for: Promising System for Selecting Healthy In Vitro–Fertilized Embryos in Cattle
Source: PLoS One. 2012 May 9;7(5):e36627. doi: 10.1371/journal.pone.0036627 (PMC3348877; doi:10.1371/journal.pone.0036627)
Supplement: Table S8 — Logistic regression analysis of variables from blastocysts (n = 111) reflecting the karyotype. (DOC) [file pone.0036627.s014.doc]

Table S8

| Variables | β^a^ | SEM^b^ | χ^2^ | *P*-value^c^ | Odds ratio | 95% C.I.^d^ |  |
| --- | --- | --- | --- | --- | --- | --- | --- |
| First cleavage: Timing | -0.405 | 0.186 | 4.761 | 0.029 | 0.667 | 0.463 to 0.960 | |
| First cleavage: 2 blastomeres | 2.539 | 0.876 | 8.398 | 0.004 | 12.667 | 2.274 to 70.550 | |
| First cleavage: Unevenness of division | -0.072 | 0.649 | 0.012 | 0.912 | 0.93 | 0.261 to 3.318 | |
| First cleavage: Presence of multiple fragments | 0.524 | 0.726 | 0.521 | 0.47 | 1.689 | 0.407 to 7.007 | |
| Second cell cycle: Duration | 0.047 | 0.33 | 0.02 | 0.887 | 1.048 | 0.548 to 2.003 | |
| Third cell cycle: Duration | -0.194 | 0.314 | 0.383 | 0.536 | 0.823 | 0.445 to 1.523 | |
| Cell cycle observed at lag-phase | 0.523 | 1.142 | 0.21 | 0.647 | 1.687 | 0.180 to 15.815 | |
| Lag-phase: Duration | -0.012 | 0.057 | 0.045 | 0.833 | 0.988 | 0.884 to 1.105 | |
| Onset of lag-phase: 4/5 blastomeres | -2.219 | 1.022 | 4.718 | 0.03 | 0.109 | 0.015 to 0.805 | |
| Onset of lag-phase: 6-8 blastomeres | 0.034 | 0.908 | 0.001 | 0.97 | 1.034 | 0.174 to 6.137 | |
| Onset of lag-phase: Unevenness of division | 0.275 | 0.617 | 0.199 | 0.656 | 1.317 | 0.393 to 4.413 | |
| Onset of lag-phase: Presence of multiple fragments | -0.145 | 0.834 | 0.03 | 0.862 | 0.865 | 0.169 to 4.434 | |
| Blastocysts at 168 hpi: Oxygen consumption | 1.007 | 0.951 | 1.122 | 0.29 | 2.737 | 0.425 to 17.643 | |

^a^ Coefficient estimate of multiple regression.

^b^ Standard error of β.

^c^ *P*-value of chi-square (χχ^2^) statistic.

^d^ 95% confidence interval.
