# Supplementary material for: Promising System for Selecting Healthy In Vitro–Fertilized Embryos in Cattle
Source: PLoS One. 2012 May 9;7(5):e36627. doi: 10.1371/journal.pone.0036627 (PMC3348877; doi:10.1371/journal.pone.0036627)
Supplement: Table S9 — Multiple regression analysis of variables from blastocysts (n = 75) reflecting gene expression. (DOC) [file pone.0036627.s015.doc]

Table S9

| Gene | Variables | β^a^ | SEM^b^ | *t*−value | *P*−value | 95% C.I.^c^ |
| --- | --- | --- | --- | --- | --- | --- |
| *IGF2R* | First cleavage: Timing | -0.068 | 0.028 | -2.450 | 0.017 | -0.124 to -0.013 |
|  | First cleavage: 2 blastomeres | 0.024 | 0.132 | 0.182 | 0.856 | -0.239 to 0.287 |
|  | First cleavage: Unevenness of division | -0.082 | 0.111 | -0.733 | 0.467 | -0.304 to 0.141 |
|  | First cleavage: Presence of multiple fragments | -0.007 | 0.117 | -0.060 | 0.953 | -0.241 to 0.227 |
|  | Second cell cycle: Duration | -0.045 | 0.051 | -0.889 | 0.378 | -0.147 to 0.057 |
|  | Third cell cycle: Duration | 0.012 | 0.032 | 0.377 | 0.708 | -0.052 to 0.077 |
|  | Cell cycle observed at lag-phase | 0.007 | 0.008 | 0.876 | 0.385 | -0.009 to 0.023 |
|  | Lag-phase: Duration | -0.102 | 0.221 | -0.463 | 0.645 | -0.544 to 0.340 |
|  | Onset of lag-phase: 4/5 blastomeres | 0.069 | 0.305 | 0.226 | 0.822 | -0.542 to 0.680 |
|  | Onset of lag-phase: 6-8 blastomeres | -0.059 | 0.211 | -0.278 | 0.782 | -0.480 to 0.363 |
|  | Onset of lag-phase: Unevenness of division | 0.097 | 0.143 | 0.678 | 0.500 | -0.189 to 0.383 |
|  | Onset of lag-phase: Presence of multiple fragments | -0.071 | 0.166 | -0.429 | 0.669 | -0.402 to 0.260 |
|  | Blastocysts at 168 hpi: Oxygen consumption | 0.174 | 0.193 | 0.901 | 0.371 | -0.212 to 0.560 |
| *IFN-tau* | First cleavage: Timing | -0.099 | 0.046 | -2.182 | 0.033 | -0.190 to -0.008 |
|  | First cleavage: 2 blastomeres | 0.488 | 0.216 | 2.261 | 0.027 | 0.056 to 0.920 |
|  | First cleavage: Unevenness of division | 0.206 | 0.186 | 1.109 | 0.272 | -0.166 to 0.578 |
|  | First cleavage: Presence of multiple fragments | -0.003 | 0.194 | -0.014 | 0.989 | -0.391 to 0.385 |
|  | Second cell cycle: Duration | -0.011 | 0.085 | -0.129 | 0.897 | -0.181 to 0.159 |
|  | Third cell cycle: Duration | 0.026 | 0.054 | 0.487 | 0.628 | -0.081 to 0.133 |
|  | Cell cycle observed at lag-phase | 0.009 | 0.013 | 0.637 | 0.526 | -0.018 to 0.035 |
|  | Lag-phase: Duration | 0.210 | 0.367 | 0.572 | 0.570 | -0.524 to0.943 |
|  | Onset of lag-phase: 4/5 blastomeres | -0.001 | 0.506 | -0.002 | 0.999 | -1.013 to1.012 |
|  | Onset of lag-phase: 6-8 blastomeres | -0.038 | 0.350 | -0.108 | 0.915 | -0.738 to 0.662 |
|  | Onset of lag-phase: Unevenness of division | 0.103 | 0.238 | 0.433 | 0.667 | -0.372 to 0.578 |
|  | Onset of lag-phase: Presence of multiple fragments | 0.087 | 0.275 | 0.317 | 0.752 | -0.463 to 0.638 |
|  | Blastocysts at 168 hpi: Oxygen consumption | 0.066 | 0.325 | 0.203 | 0.840 | -0.583 to 0.715 |
| *AKR1B1* | First cleavage: Timing | -0.075 | 0.046 | -1.640 | 0.106 | -0.167 to 0.016 |
|  | First cleavage: 2 blastomeres | 0.058 | 0.216 | 0.269 | 0.789 | -0.374 to 0.491 |
|  | First cleavage: Unevenness of division | -0.016 | 0.183 | -0.089 | 0.930 | -0.383 to 0.350 |
|  | First cleavage: Presence of multiple fragments | 0.880 | 0.193 | 4.566 | < 0.001 | 0.495 to 1.266 |
|  | Second cell cycle: Duration | 0.085 | 0.084 | 1.009 | 0.317 | -0.083 to 0.252 |
|  | Third cell cycle: Duration | -0.001 | 0.053 | -0.010 | 0.992 | -0.107 to 0.106 |
|  | Cell cycle observed at lag-phase | 0.011 | 0.013 | 0.806 | 0.424 | -0.016 to 0.037 |
|  | Lag-phase: Duration | -0.108 | 0.364 | -0.298 | 0.767 | -0.836 to 0.619 |
|  | Onset of lag-phase: 4/5 blastomeres | 0.303 | 0.503 | 0.604 | 0.548 | -0.702 to1.308 |
|  | Onset of lag-phase: 6-8 blastomeres | 0.227 | 0.347 | 0.655 | 0.515 | -0.466 to 0.921 |
|  | Onset of lag-phase: Unevenness of division | 0.049 | 0.235 | 0.208 | 0.836 | -0.422 to 0.520 |
|  | Onset of lag-phase: Presence of multiple fragments | -0.011 | 0.272 | -0.039 | 0.969 | -0.555 to 0.534 |
|  | Blastocysts at 168 hpi: Oxygen consumption | -0.128 | 0.318 | -0.403 | 0.689 | -0.763 to 0.507 |
| *PLAC8* | First cleavage: Timing | -0.005 | 0.033 | -0.142 | 0.888 | -0.071 to 0.061 |
|  | First cleavage: 2 blastomeres | 0.047 | 0.156 | 0.303 | 0.763 | -0.265 to 0.359 |
|  | First cleavage: Unevenness of division | 0.015 | 0.132 | 0.117 | 0.908 | -0.249 to 0.280 |
|  | First cleavage: Presence of multiple fragments | -0.015 | 0.139 | -0.106 | 0.916 | -0.293 to 0.263 |
|  | Second cell cycle: Duration | 0.036 | 0.060 | 0.591 | 0.557 | -0.085 to 0.157 |
|  | Third cell cycle: Duration | -0.008 | 0.038 | -0.218 | 0.828 | -0.085 to 0.068 |
|  | Cell cycle observed at lag-phase | 0.001 | 0.010 | 0.108 | 0.914 | -0.018 to 0.020 |
|  | Lag-phase: Duration | -0.115 | 0.262 | -0.437 | 0.664 | -0.639 to 0.410 |
|  | Onset of lag-phase: 4/5 blastomeres | -0.252 | 0.362 | -0.697 | 0.489 | -0.977 to 0.472 |
|  | Onset of lag-phase: 6-8 blastomeres | 0.009 | 0.250 | 0.034 | 0.973 | -0.492 to 0.509 |
|  | Onset of lag-phase: Unevenness of division | 0.201 | 0.170 | 1.182 | 0.242 | -0.139 to 0.540 |
|  | Onset of lag-phase: Presence of multiple fragments | 0.077 | 0.196 | 0.394 | 0.695 | -0.316 to 0.470 |
|  | Blastocysts at 168 hpi: Oxygen consumption | 0.731 | 0.229 | 3.193 | 0.002 | 0.273 to 1.189 |
| *CDX2* | First cleavage: Timing | -0.023 | 0.046 | -0.510 | 0.612 | -0.115 to 0.069 |
|  | First cleavage: 2 blastomeres | 0.340 | 0.218 | 1.557 | 0.125 | -0.097 to 0.776 |
|  | First cleavage: Unevenness of division | 0.042 | 0.188 | 0.225 | 0.822 | -0.334 to 0.418 |
|  | First cleavage: Presence of multiple fragments | 0.088 | 0.196 | 0.450 | 0.655 | -0.304 to 0.480 |
|  | Second cell cycle: Duration | -0.085 | 0.086 | -0.993 | 0.325 | -0.257 to 0.086 |
|  | Third cell cycle: Duration | 0.001 | 0.054 | 0.019 | 0.985 | -0.107 to 0.109 |
|  | Cell cycle observed at lag-phase | 0.007 | 0.013 | 0.485 | 0.630 | -0.020 to 0.034 |
|  | Lag-phase: Duration | -0.209 | 0.371 | -0.565 | 0.574 | -0.951 to 0.532 |
|  | Onset of lag-phase: 4/5 blastomeres | -0.007 | 0.512 | -0.013 | 0.990 | -1.030 to 1.017 |
|  | Onset of lag-phase: 6-8 blastomeres | 0.152 | 0.354 | 0.429 | 0.670 | -0.556 to 0.859 |
|  | Onset of lag-phase: Unevenness of division | 0.114 | 0.240 | 0.473 | 0.638 | -0.367 to 0.594 |
|  | Onset of lag-phase: Presence of multiple fragments | 0.331 | 0.278 | 1.189 | 0.239 | -0.226 to 0.887 |
|  | Blastocysts at 168 hpi: Oxygen consumption | -0.002 | 0.328 | -0.006 | 0.995 | -0.658 to 0.654 |

^a^ Coefficient estimate of multiple regression.

^b^ Standard error of β.

^c^ 95% confidence interval.
