# Supplementary material for: Promising System for Selecting Healthy In Vitro–Fertilized Embryos in Cattle
Source: PLoS One. 2012 May 9;7(5):e36627. doi: 10.1371/journal.pone.0036627 (PMC3348877; doi:10.1371/journal.pone.0036627)
Supplement: Table S10 — Estimated probabilities derived from the fitted logistic regression model of pregnancy success. (DOC) [file pone.0036627.s016.doc]

Table S10

| End of the first cleavage | | | High oxygen consumption at blastocyst stage (1) ^b^ | |  | Low oxygen consumption at blastocyst stage (0) ^b^ | |
| --- | --- | --- | --- | --- | --- | --- | --- |
|  |  |  | Number of blastomeres at the onset of the lag-phase | |  | Number of blastomeres at the onset of the lag-phase | |
| Timing ^a^ | Number of blastomeres | Multiple fragments | 4/5 (0) | 6–16 (1) |  | 4/5 (0) | 6–16 (1) |
| Fast (1) | 2 (1) | Absence (0) | 0.44 | 0.82 |  | 0.04 | 0.21 |
|  |  | Presence (1) | 0.14 | 0.50 |  | 0.01 | 0.05 |
|  | 3/4 (0) | Absence (0) | 0.09 | 0.37 |  | 0.00 | 0.03 |
|  |  | Presence (1) | 0.02 | 0.11 |  | 0.00 | 0.01 |
| Slow (0) | 2 (1) | Absence (0) | 0.08 | 0.34 |  | 0.00 | 0.03 |
|  |  | Presence (1) | 0.02 | 0.10 |  | 0.00 | 0.01 |
|  | 3/4 (0) | Absence (0) | 0.01 | 0.06 |  | 0.06 | 0.00 |
|  |  | Presence (1) | 0.00 | 0.01 |  | 0.00 | 0.00 |

The table was created with data from 52 blastocysts.

The nominal variable of each prognostic factor is shown in parentheses.

Probability estimates of selected combinations of prognostic factors were calculated using a full logistic regression model according to the formula $\frac{1}{1+e^{-z}}$ where *e* is the base of the natural logarithm, 2.718, and z = −7.364 + 2.061 × [timing of first cleavage] + 2.214 × [number of blastomeres at first cleavage] + -1.547 × [presence or absence of multiple fragments] + 1.789 × [number of blastomeres at the onset of the lag-phase] + 2.844 × [oxygen consumption of the blastocyst at 168 hpi]. Each prognostic factor in brackets was assigned a dummy variable of 1 or 0.

^a^ Fast and slow for the first cleavage were defined as ≤27.0 and >27.0 hpi, respectively.

^b^ High and low oxygen consumption were defined as <0.84 and ≥0.84 × 10^–14^ mol s^–1^, respectively.
